# Association between the COVID-19 outbreak and opioid prescribing by U.S. dentists

**DOI:** 10.1371/journal.pone.0293621

**Published:** 2023-11-02

**Authors:** Jason Zhang, Romesh P. Nalliah, Jennifer F. Waljee, Chad M. Brummett, Kao-Ping Chua

**Affiliations:** 1 Department of Pediatrics, Susan B. Meister Child Health Evaluation and Research Center, University of Michigan Medical School, Ann Arbor, MI, United States of America; 2 University of Michigan School of Dentistry, Ann Arbor, MI, United States of America; 3 Michigan Opioid Prescribing Engagement Network, University of Michigan Medical School, Ann Arbor, MI, United States of America; 4 Section of Plastic Surgery, Department of Surgery, University of Michigan Medical School, Ann Arbor, MI, United States of America; 5 Division of Pain Medicine, Department of Anesthesiology, University of Michigan Medical School, Ann Arbor, MI, United States of America; Hosei University: Hosei Daigaku, JAPAN

## Abstract

**Background:**

U.S. data on opioid prescribing by dentists are limited to 2019. More recent data are needed to understand the effect of the COVID-19 outbreak on dental opioid prescribing, characterize current practices, and determine if dental opioid stewardship initiatives are still warranted.

**Objective:**

To evaluate the association between the COVID-19 outbreak and the rate of opioid prescribing by U.S. dentists

**Methods:**

During February—April 2023, the authors conducted a cross-sectional analysis of the IQVIA Longitudinal Prescription Database, which reports 92% of prescriptions dispensed in U.S. retail pharmacies. The authors calculated the monthly dental opioid dispensing rate, defined as the monthly number of dispensed opioid prescriptions from dentists per 100,000 U.S. individuals, during January 2016-February 2020 and June 2020-December 2022. To prevent distortions in trends, data from March–May 2020, when dental opioid dispensing declined sharply, were excluded. Using linear segmented regression models, the authors assessed for level and slope changes in the dental opioid dispensing rate during June 2020.

**Results:**

Analyses included 81,189,605 dental opioid prescriptions. The annual number of prescriptions declined from 16,105,634 in 2016 to 8,910,437 in 2022 (-44.7%). During January 2016-February 2020, the dental opioid dispensing rate declined -3.9 (95% CI: -4.3, -3.6) per month. In June 2020, this rate abruptly increased by 31.4 (95% CI: 19.3, 43.5) and the monthly decline in the dental opioid dispensing rate slowed to -2.1 (95% CI: -2.6, -1.6) per month. As a result, 6.1 million more dental opioid prescriptions were dispensed during June 2020-December 2022 than would be predicted had trends during January 2016-February 2020 continued.

**Discussion:**

U.S. dental opioid prescribing is declining, but the rate of this decline slowed after the COVID-19 outbreak. Findings highlight the continued importance of dental opioid stewardship initiatives.

## Introduction

Evidence suggests that non-opioid medications are equally effective for analgesia for most routine dental procedures [1–4]. Despite this, opioid prescribing after these procedures remains common in the U.S [5–10]. Dental opioid prescribing rates are much higher in the U.S. than other countries; in 2016, dentists in the U.S. prescribed opioids at 37 times the rate for dentists in England [11, 12]. U.S. dentists account for 40% of opioid prescriptions to adolescents and young adults, a vulnerable population with high rates of opioid misuse [13–18]. High rates of dental opioid prescribing in the U.S. are concerning, as this prescribing is associated with a variety of opioid-related harms, including diversion, opioid use disorder, and opioid overdose [19–26].

National data indicate that the rate of U.S. dental opioid prescribing per capita increased until 2016 and subsequently decreased through 2019 [8, 27], potentially owing to dental opioid stewardship initiatives and heightened societal awareness regarding the harms of opioids [10, 28–32]. However, more recent national data are unavailable. This is an important gap because opioid prescribing patterns by dentists may have changed since 2019, particularly in light of the COVID-19 pandemic. For example, the outbreak of COVID-19 in March 2020 was associated with an initial decrease in the number of dental procedures [33], which would tend to decrease the population-level rate of exposure to dental opioid prescriptions. On the other hand, dentists may have been more likely to prescribe opioids after procedures during the pandemic owing to barriers to scheduling routine care and follow-up visits for uncontrolled pain, which would tend to increase the population-level rate of exposure to dental opioid prescriptions. Furthermore, in cases where dental procedures for acutely painful dental emergencies were not possible due to pandemic-related restrictions, opioid prescribing may have increased to serve as a stopgap until dental care resumed. Such stopgap prescribing may have been facilitated by the increased use of teledentistry during the pandemic.

In this study, the authors analyzed national prescription dispensing data from January 2016 to December 2022 to evaluate the association between the COVID-19 outbreak and the rate of dispensed dental opioid prescriptions to U.S. patients, both overall and among key demographic subgroups.

## Methods

### Study sample

During February—April 2023, the authors conducted a cross-sectional analysis of the 2016–2022 IQVIA Longitudinal Prescription Database, a prescription-level database representing 92% of prescriptions dispensed from U.S. retail pharmacies across all payers, including cash. The data are not projected to national totals. The database includes an encrypted patient identifier and data on opioid name, quantity, days supplied, dispensing date, and prescriber specialty. No data on race/ethnicity, household income, or indication for prescriptions are reported. Because the data were de-identified administrative data that have already been collected, the Institutional Review Board of the University of Michigan Medical School did not regulate this study as human subjects research; consequently, informed consent was not required.

Opioid prescriptions were identified using IQVIA’s market definitions of these medications. This definition excludes opioid cough-and-cold medications and opioids used to treat opioid use disorder. However, it includes liquid formulations as well as non-oral formulations, such as patches. Analyses included opioid prescriptions written by general dentists, dental subspecialists, or oral and maxillofacial surgeons to patients residing in one of the 50 U.S. states or the District of Columbia. Prescriptions with invalid or missing data for days supplied, dispensed quantity, or strength were excluded.

### Measures

The primary outcome was the monthly dental opioid dispensing rate, defined as the monthly number of dispensed opioid prescriptions written by dentists per 100,000 U.S. individuals of all ages. Population denominators were derived from the U.S. Census Bureau (S2 Table). This outcome was calculated overall and among subgroups defined by age group (0–11, 12–25, 26–44, 45–64, and ≥65 years old), sex (male/female), method of payment (commercial, Medicaid, Medicare, cash), U.S. Census region (Northeast, Midwest, South, West), and specialty (oral and maxillofacial surgeon versus general dentist or dental subspecialist). For the subgroup analyses by age group, sex, method of payment, and Census region, we used population denominators for the subgroup in question (e.g., for patients aged 12–25 years, the outcome was the number of dispensed opioid prescriptions from dentists per 100,000 U.S. individuals aged 12–25 years). For the subgroup analysis by specialty, the population denominator was U.S. individuals of all ages, as it was in the overall analysis. Additional details are included in S2 Table.

To determine whether changes in the monthly dental opioid dispensing rate might be influenced by changes in the mean duration or size of dental opioid prescriptions (e.g., the provision of fewer but larger opioid prescriptions), the authors also assessed monthly mean days supplied per prescription and mean total morphine milligram equivalents (MMEs) per prescription, a standardized measure of opioid prescription size. Mean total MMEs were calculated by multiplying quantity, strength per dose, and MME conversion factors from the Centers for Disease Control and Prevention [1].

### Statistical analysis

The authors assessed changes in trends in the monthly dental opioid dispensing rate between two periods: January 2016-February 2020 and June 2020-December 2022. To avoid distortions in analyses of trends, data from March-May 2020, a period during which the dental opioid dispensing date declined sharply owing to pandemic-related postponement of dental procedures, were excluded [33]. Using an interrupted time series design, the authors fitted linear segmented regression models assessing for abrupt slope or level changes in the monthly dental opioid dispensing rate during June 2020. To facilitate interpretation, the authors calculated the difference between the cumulative number of dental opioid prescriptions that were dispensed from June 2020-December 2022 and the number that would have been dispensed during this period had trends from January 2016-February 2020 continued.

In all models, the authors assessed for autocorrelation and employed robust Newey-West standard errors with the appropriate number of lags. Analyses used two-sided hypothesis test with α = 0.05 and were conducted using SAS 9.4, Stata 17.1/SE, and R (version 4.2.2).

## Results

### Sample characteristics

The sample initially included 84,080,453 dental opioid prescriptions for 54,699,674 patients. The authors excluded 966,804 (1.1%) prescriptions owing to missing or invalid dosing data or residence outside of one of the 50 U.S. states or the District of Columbia. The authors additionally excluded 1,924,044 (2.3%) prescriptions dispensed in March-May 2020. After these exclusions, the sample included 81,189,605 dental opioid prescriptions for 53,182,495 patients. Mean (SD) age at the time of the earliest fill during the study period was 42.3 (19.0) years; 28,470,645 (53.5%) patients were male. Characteristics of patients with dispensed opioid prescriptions from U.S. dentists each year are shown in Table 1.

**Table 1:** Characteristics of patients with dispensed opioid prescriptions from U.S. dentists.

Characteristic	2016	2017	2018	2019	2020	2021	2022
**# patients**	12,314,344	11,527,064	10,127,550	8,964,581	6,653,670	8,165,215	7,444,966
**Age group**							
0–11 years	188,524 (1.5%)	137,820 (1.2%)	81,358 (0.8%)	55,133 (0.6%)	34,773 (0.5%)	38,763 (0.5%)	32,575 (0.4%)
12–25 years	2,555,813 (20.8%)	2,412,345 (20.9%)	2,151,618 (21.2%)	1,935,918 (21.6%)	1,431,103 (21.5%)	1,743,481 (21.4%)	1,617,122 (21.7%)
26–44 years	3,845,235 (31.2%)	3,579,638 (31.1%)	3,128,263 (30.9%)	2,707,886 (30.2%)	1,987,274 (29.9%)	2,385,648 (29.2%)	2,053,834 (27.6%)
45–64 years	3,981,897 (32.3%)	3,671,738 (31.9%)	3,193,793 (31.5%)	2,783,714 (31.1%)	2,047,436 (30.8%)	2,462,052 (30.2%)	2,220,471 (29.8%)
65 years and above	1,544,524 (12.5%)	1,531,409 (13.3%)	1,425,932 (14.1%)	1,348,435 (15.0%)	1,053,869 (15.8%)	1,408,712 (17.3%)	1,399,157 (18.8%)
**Sex**							
Male	5,634,581 (45.8%)	5,281,523 (45.8%)	4,657,180 (46.0%)	4,124,202 (46.0%)	3,037,295 (45.6%)	3,757,403 (46.0%)	3,470,667 (46.6%)
Female	6,624,983 (53.8%)	6,193,987 (53.7%)	5,454,438 (53.9%)	4,828,207 (53.9%)	3,614,349 (54.3%)	4,405,840 (54.0%)	3,973,448 (53.4%)
Unknown	54,780 (0.4%)	51,554 (0.4%)	15,932 (0.2%)	12,172 (0.1%)	2,026 (0.0%)	1,972 (0.0%)	851 (0.0%)
**Census region**							
Northeast	1,689,919 (13.7%)	1,441,434 (12.5%)	1,215,910 (12.0%)	1,051,629 (11.7%)	744,962 (11.2%)	924,530 (11.3%)	842,960 (11.3%)
Midwest	2,512,832 (20.4%)	2,378,340 (20.6%)	2,042,601 (20.2%)	1,785,825 (19.9%)	1,333,006 (20.0%)	1,660,512 (20.3%)	1,515,796 (20.4%)
South	5,423,538 (44.0%)	5,172,082 (44.9%)	4,610,820 (45.5%)	4,144,709 (46.2%)	3,090,890 (46.5%)	3,717,136 (45.5%)	3,436,451 (46.2%)
West	2,688,055 (21.8%)	2,535,208 (22.0%)	2,258,219 (22.3%)	1,982,418 (22.1%)	1,484,812 (22.3%)	1,863,037 (22.8%)	1,649,759 (22.2%)
**Method of payment** ^**a**^							
Cash	1,297,926 (10.5%)	1,138,083 (9.9%)	980,848 (9.7%)	804,232 (9.0%)	595,687 (9.0%)	743,052 (9.1%)	599,690 (8.1%)
Medicaid	1,880,239 (15.3%)	1,755,822 (15.2%)	1,462,048 (14.4%)	1,217,423 (13.6%)	885,886 (13.3%)	1,121,579 (13.7%)	1,036,017 (13.9%)
Medicare	1,326,368 (10.8%)	1,320,778 (11.5%)	1,217,147 (12.0%)	1,144,118 (12.8%)	915,101 (13.8%)	1,195,576 (14.6%)	1,189,884 (16.0%)
Commercial	7,808,833 (63.4%)	7,312,133 (63.4%)	6,467,307 (63.9%)	5,798,684 (64.7%)	4,256,823 (64.0%)	5,104,330 (62.5%)	4,619,158 (62.0%)

^a^Represents method of payment for the first opioid prescription fill during the year

Among the 81,189,605 prescriptions, the most common method of payment was commercial insurance (51,024,628; 62.8%). Overall, 58,840,579 (72.5%) of opioid prescriptions were prescribed by general dentists or dental subspecialists, while 22,349,026 (27.5%) were prescribed by oral and maxillofacial surgeons. The 3 most common opioids prescribed were hydrocodone (49,163,654; 60.6%), codeine (18,547,695; 22.8%), and oxycodone (8,678,172; 10.7%).

### Trends in the monthly dental opioid dispensing rate

**Fig 1:**
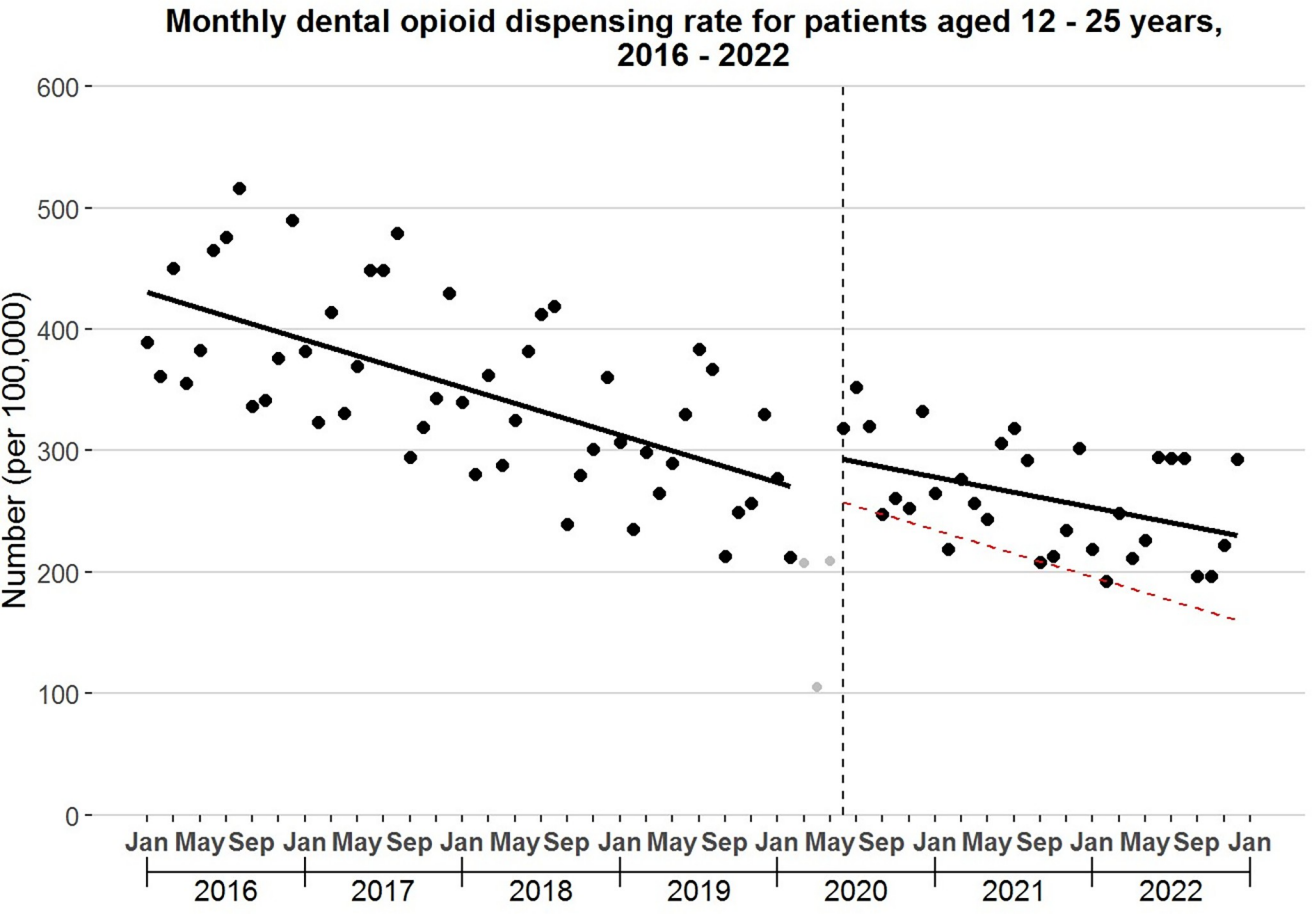
Monthly dental opioid dispensing rate, 2016–2022. This rate is defined as the monthly number of dispensed opioid prescriptions from dentists per 100,000 U.S. individuals of all ages. The black lines are fitted lines from segmented regression models assessing for abrupt level or slope changes in June 2020 (vertical line). The red dashed line is the counterfactual trend, representing the trend that would have occurred had trends from January 2016-February 2020 continued.

In 2016, 16,105,634 dental opioid prescriptions were dispensed, compared with 8,910,437 in 2022, representing a 44.7% decline. As shown in Fig 1, the rate of decline varied over time. During January 2016-February 2020, the monthly dental opioid dispensing rate (i.e., the monthly number of dispensed opioid prescriptions from dentists per 100,000 U.S. individuals) declined -3.9 (95% CI: -4.3,-3.6) per month. This rate increased abruptly by 31.4 (95% CI: 19.3, 43.5) in June 2020. From June 2020-December 2022, the monthly rate of decline in the dental opioid dispensing rate slowed to -2.1 (95% CI: -2.6,-1.6) per month. During this latter period, 25.0 million dental opioid prescriptions were dispensed, but if trends from January 2016-February 2020 had continued, this number would have been 18.9 million. Consequently, 6.1 million more dental opioid prescriptions were dispensed during June 2020-December 2022 than was expected, representing a 32.0% increase (Table 2).

**Table 2:** Segmented regression model coefficients for dental opioid prescription dispensing rate.

	Segmented regression model coefficients, # prescriptions/100,000 U.S. individuals^a^					Cumulative # excess dental opioid prescriptions dispensed from June 2020-December 2022 (% difference)
	Intercept [95% CI]	Change per month before February 2020 [95% CI]	Level change during June 2020 [95% CI]	Slope change after June 2020 [95% CI]	Change per month after June 2020 [95% CI]	
**All prescriptions**	444.7 [433.7, 455.6]	-3.9 [-4.3, -3.6]	31.4 [19.3, 43.5]	1.8 [1.3, 2.4]	-2.1 [-2.6, -1.6]	6,050,918 (32.0%)
**Age group**						
0–11 years	39.6 [37.7, 41.6]	-0.7 [-0.8, -0.6]	4.9 [2.8, 7]	0.6 [0.5, 0.7]	-0.1 [-0.1, -0.1]	104,855 (1430.3%)
12–25 years	433.4 [399.2, 467.7]	-3.3 [-4.4, -2.1]	25.3 [-19.4, 70]	1.2 [-0.9, 3.2]	-2.1 [-4, -0.2]	793,460 (19.5%)
26–44 years	601 [577.9, 624.1]	-6.1 [-6.7, -5.4]	49.1 [31.4, 66.7]	2.3 [1.5, 3.1]	-3.8 [-4.3, -3.4]	2,161,155 (41.7%)
45–64 years	561.1 [547.8, 574.5]	-5 [-5.4, -4.6]	37.1 [26.8, 47.4]	2.4 [1.9, 2.9]	-2.6 [-2.9, -2.3]	1,892,386 (31.6%)
65 years and above	353.9 [344.5, 363.3]	-2.2 [-2.5, -2]	21.8 [7, 36.6]	1.6 [0.9, 2.4]	-0.6 [-1.3, 0.1]	810,801 (22.7%)
**Sex**						
Male	405.5 [395.5, 415.4]	-3.5 [-3.8, -3.2]	23.9 [13.7, 34.2]	1.9 [1.4, 2.3]	-1.6 [-2, -1.3]	2,633,328 (30.0%)
Female	478.7 [466.4, 491.1]	-4.3 [-4.6, -3.9]	38.4 [24, 52.8]	1.7 [1.1, 2.4]	-2.5 [-3.1, -2]	3,339,092 (32.7%)
**Census region**						
Northeast	325 [314.5, 335.6]	-3.5 [-3.9, -3.2]	26.9 [16.6, 37.3]	2.4 [2, 2.8]	-1.1 [-1.4, -0.9]	1,115,921 (69.2%)
Midwest	430 [418.1, 441.8]	-3.9 [-4.3, -3.6]	34.7 [23, 46.5]	2.1 [1.5, 2.6]	-1.9 [-2.3, -1.5]	1,398,074 (38.7%)
South	529.9 [516, 543.8]	-4.4 [-4.8, -4]	31.4 [13.4, 49.3]	1.7 [0.9, 2.5]	-2.7 [-3.5, -2]	2,235,432 (23.8%)
West	412.5 [403.4, 421.6]	-3.5 [-3.8, -3.3]	31.4 [21.9, 40.9]	1.4 [1, 1.9]	-2.1 [-2.4, -1.8]	1,290,599 (29.7%)
**Method of payment**						
Cash	621.3 [580.8, 661.9]	-5.3 [-6.5, -4.1]	43.1 [9.6, 76.5]	0.7 [-0.8, 2.3]	-4.6 [-5.6, -3.5]	356,716 (19.9%)
Medicaid	414.4 [402, 426.8]	-4.6 [-5, -4.3]	28.6 [17.6, 39.6]	2 [1.5, 2.6]	-2.6 [-3.1, -2.2]	1,254,668 (57.2%)
Medicare	303.8 [296.6, 311]	-2 [-2.2, -1.7]	16.3 [5.8, 26.8]	1.7 [1.2, 2.1]	-0.3 [-0.7, 0.1]	731,812 (23.5%)
Commercial	471.2 [462.1, 480.4]	-4 [-4.3, -3.7]	34.2 [22.2, 46.1]	1.9 [1.3, 2.5]	-2.1 [-2.6, -1.6]	3,573,110 (29.8%)
**Prescriber type**						
Oral/maxillofacial surgeons	106.4 [103.4, 109.5]	-0.7 [-0.7, -0.6]	6.5 [2.3, 10.7]	0.3 [0.2, 0.5]	-0.3 [-0.5, -0.1]	1,194,975 (18.4%)
Other dentists	338.2 [330, 346.5]	-3.3 [-3.5, -3.1]	24.9 [18.1, 31.7]	1.5 [1.2, 1.8]	-1.8 [-2, -1.6]	4,855,943 (39.0%)

^a^For the subgroup analyses by age group, sex, method of payment, and Census region, we used population denominators for the subgroup in question (e.g., for patients aged 12–25 years, the outcome was the number of dispensed opioid prescriptions from dentists per 100,000 U.S. individuals aged 12–25 years). For the subgroup analysis by specialty, the population denominator was U.S. individuals of all ages, as it was in the overall analysis. Additional details are included in S2 Table.

### Subgroup analyses

As in the main analysis, there was a level increase and slope increase (i.e., slowing of the pre-existing decline) during June 2020 in each age group, except for patients aged 12–25 years, for which there was neither a level nor a slope change (S1 Fig). There was also heterogeneity in changes during June 2020 by method of payment (S2 Fig). The cumulative number of dental opioid prescriptions dispensed to Medicaid patients during June 2020-December 2022 was 57.2% higher than predicted based on trends from January 2016-February 2020, compared with 29.8% and 23.5% higher than predicted for the commercially insured and Medicare populations, respectively. This indicates that the degree of slowing in the pre-existing decline in dental opioid prescribing rate was greater in the Medicaid population.

Changes in the dental opioid dispensing rate during June 2020 varied little by patient sex (S3 Fig) but varied substantially among Census regions (S4 Fig). In the Northeast, the cumulative number of dental opioid prescriptions dispensed during June 2020-December 2022 was 69.2% higher than predicted, compared with 38.7%, 29.7%, and 23.8% higher in the Midwest, West, and South (Table 2).

Changes in the dental opioid dispensing rate during June 2020 also varied by dental specialty (S5 Fig). For oral and maxillofacial surgeons, there was only a modest slowing of the pre-existing decline in the dental opioid dispensing rate during June 2020, such that the cumulative number of dispensed opioid prescriptions from oral and maxillofacial surgeons during June 2020-December 2022 was 18.4% higher than predicted based on trends from January 2016-February 2020. In contrast, for general dentists and dental subspecialists, the corresponding quantity was 39.0% higher than predicted.

### Mean total MMEs and mean days supplied per prescription

Monthly mean total MMEs per prescription and monthly mean days supplied per prescription declined from January 2016-February 2020. For mean total MMEs, there was no level change in June 2020 but there was a slope increase; the slope after June 2020 was -0.2 (95% CI: -0.21, -0.18) MMEs per month. For mean days supplied, there was no level change in June 2020 but there was a small slope increase; the slope from June 2020-December 2022 was flat (S6 and S7 Figs and S3 Table).

## Discussion

To the authors’ knowledge, this study provides the most recent national data on U.S. dental opioid prescribing and is the first to capture changes in this prescribing associated with the COVID-19 outbreak. Findings indicate that the annual number of dispensed dental opioid prescriptions declined 44.7% between 2016 and 2022. However, the rate of decline varied substantially over time. The sharpest decline in the dental opioid dispensing rate occurred between January 2016 and February 2020. After June 2020, this rate continued to decline, but at a slower pace than before June 2020. As a result of this slowing, 6.1 million more dental opioid prescriptions were dispensed during June 2020-December 2022 than would be predicted had trends from January 2016-February 2020 continued.

Collectively, this study provides both reasons for optimism and concern. On the one hand, findings suggest that large reductions in dental opioid prescribing were achieved between 2016 and 2022. On the other hand, findings also suggest that progress in reducing dental opioid prescribing slowed after the COVID-19 outbreak, highlighting the importance of continuing to invest in efforts to monitor and improve the appropriateness of dental opioid prescribing.

The reason for the slowing in the rate of decline in dental opioid prescribing warrants further investigation. Potential explanations might include a backlash against efforts to reduce opioid prescribing in the U.S. or increases in “just-in-case” opioid prescribing at discharge from dental procedures owing to concerns about accessing opioids during the pandemic [34]. It is also possible that worsened access to dental care during the pandemic increased the prevalence of acutely painful dental emergencies, leading to an increase in the number of dental opioid prescriptions written for these emergencies. In potential support of this hypothesis, the decline in dental opioid prescribing slowed particularly sharply after June 2020 in the Medicaid population, which faces high barriers to accessing dental care [35, 36].

A final potential explanation is that dental opioid prescribing is reaching a natural plateau and that any further reductions in prescribing would be infeasible without worsening pain control. However, this possibility seems unlikely, given that the majority of dental opioid prescribing is for routine procedures for which non-opioid analgesics are safer and equally effective options for analgesia, such as tooth extraction.[2–4] As further evidence against the notion that reductions in U.S. dental opioid prescribing are no longer possible, U.S. dentists accounted for 215 opioid prescriptions per 100,000 people in December 2022. In contrast, a prior study found that dentists in England accounted for 50 opioid prescriptions per 100,000 people in 2016, over 4 times lower.[11]

Subgroup analyses revealed heterogeneity in changes in the dental opioid dispensing rate. In contrast to other age groups, there was no level or slope change in this rate during June 2020 among adolescents and young adults aged 12–25 years, and the dental opioid dispensing rate in this age group continued to decline after June 2020. These trends are reassuring, as dentists account for over 40% of opioid prescriptions to adolescents and young adults and as 80% of dental opioid prescriptions in this age group is for tooth extraction.[18] There was also substantial geographic variability in the degree to which the decline in dental opioid prescribing slowed after June 2020, with the greatest slowing occurring in the Northeast. Future research using dental claims will be needed to investigate whether these regional differences were driven by changes in dental procedure volume, changes in the rate of opioid prescribing after procedures, and/or changes in the severity of dental conditions.

Our findings suggest that dental opioid stewardship initiatives may be important to implement during future pandemics. As most dental opioid prescribing occurs in the community, such initiatives should derive from sources with broad community reach, such as the American Dental Association (ADA). The ADA has the influence to reinforce the importance of adhering to the basic principles of dental opioid stewardship during pandemics, including promoting use of multimodal non-opioid analgesia as first-line therapy and querying prescription drug monitoring programs when appropriate. During pandemics, the ADA could also work to ensure that patients and non-dentist clinicians who care for acute dental emergencies (e.g., emergency medicine physicians) are aware of the benefits of non-opioids for the management of most dental pain. Finally, when social distancing measures are necessary, policymakers should take steps to maintain access to dental care, thus avoiding the need for opioid prescribing for complications associated with postponement of routine dental care. These steps include ensuring that dentists have sufficient access to personal protective equipment.

The primary strength of this study was the use of timely data from an all-payer national database. However, the study also had limitations. First, the database only included dispensed prescriptions. Consequently, this study likely underestimates the rate of dental opioid prescribing. Second, the database lacks information on the procedures for which opioid prescriptions were written. Finally, the dental opioid dispensing rate would be an imperfect measure of prevalence if the mean size or duration of dental opioid prescriptions changed. However, sensitivity analyses revealed that these parameters were essentially flat after June 2020, suggesting that changes in the dental opioid dispensing rate after this month were not artificially driven by changes in the size or duration of opioid prescriptions.

## Conclusion

Opioid prescribing by U.S. dentists decreased markedly between 2016 and 2022, but the rate of decline slowed substantially after the COVID-19 outbreak. Future research should continue to monitor dental opioid prescribing trends beyond 2022 to evaluate whether this slowing continues. In the meantime, renewed investment in opioid stewardship efforts is needed to ensure that the contribution of dental opioid prescribing to the U.S. opioid epidemic continues to be mitigated.

## Supporting information

S1 Checklist (DOCX)

S1 Fig Monthly dental opioid dispensing rate by age group, 2016–2022. This rate is defined as the monthly number of dispensed opioid prescriptions from dentists per 100,000 U.S. individuals of all ages. The black lines are fitted lines from segmented regression models assessing for abrupt level or slope changes in June 2020 (vertical line). The red dashed line is the counterfactual trend, representing the trend that would have occurred had trends from January 2016-February 2020 continued.
(DOCX)

S2 Fig Monthly dental opioid dispensing rate by method of payment, 2016–2022. This rate is defined as the monthly number of dispensed opioid prescriptions from dentists per 100,000 U.S. individuals of all ages. The black lines are fitted lines from segmented regression models assessing for abrupt level or slope changes in June 2020 (vertical line). The red dashed line is the counterfactual trend, representing the trend that would have occurred had trends from January 2016-February 2020 continued.
(DOCX)

S3 Fig Monthly dental opioid dispensing rate by sex, 2016–2022. This rate is defined as the monthly number of dispensed opioid prescriptions from dentists per 100,000 U.S. individuals of all ages. The black lines are fitted lines from segmented regression models assessing for abrupt level or slope changes in June 2020 (vertical line). The red dashed line is the counterfactual trend, representing the trend that would have occurred had trends from January 2016-February 2020 continued.
(DOCX)

S4 Fig Monthly dental opioid dispensing rate by region, 2016–2022. This rate is defined as the monthly number of dispensed opioid prescriptions from dentists per 100,000 U.S. individuals of all ages. The black lines are fitted lines from segmented regression models assessing for abrupt level or slope changes in June 2020 (vertical line). The red dashed line is the counterfactual trend, representing the trend that would have occurred had trends from January 2016-February 2020 continued.
(DOCX)

S5 Fig Monthly dental opioid dispensing rate by specialty, 2016–2022. This rate is defined as the monthly number of dispensed opioid prescriptions from dentists per 100,000 U.S. individuals of all ages. The black lines are fitted lines from segmented regression models assessing for abrupt level or slope changes in June 2020 (vertical line). The red dashed line is the counterfactual trend, representing the trend that would have occurred had trends from January 2016-February 2020 continued.
(DOCX)

S6 Fig Monthly mean total MMEs for dental opioid prescriptions, 2016–2022. The black lines are fitted lines from segmented regression models assessing for abrupt level or slope changes in June 2020 (vertical line). The red dashed line is the counterfactual trend, representing the trend that would have occurred had trends from January 2016-February 2020 continued.
(DOCX)

S7 Fig Monthly mean days supplied for dental opioid prescriptions, 2016–2022. The black lines are fitted lines from segmented regression models assessing for abrupt level or slope changes in June 2020 (vertical line). The red dashed line is the counterfactual trend, representing the trend that would have occurred had trends from January 2016-February 2020 continued.
(DOCX)

S1 Table Opioids in the IQVIA data. (DOCX)

S2 Table U.S. Census population denominators, 2016–2022. (DOCX)

S3 Table Segmented regression model coefficients for mean total MME and mean days supplied per dental opioid prescription. (DOCX)
