# Supplementary material for: Association between the COVID-19 outbreak and opioid prescribing by U.S. dentists
Source: PLoS One. 2023 Nov 2;18(11):e0293621. doi: 10.1371/journal.pone.0293621 (PMC10621808; doi:10.1371/journal.pone.0293621)
Supplement: S1 Checklist — (DOCX) [file pone.0293621.s001.docx]

STROBE Statement—checklist of items that should be included in reports of observational studies

|  | Item No. | Recommendation | Page  No. | Relevant text from manuscript |
| --- | --- | --- | --- | --- |
| **Title and abstract** | 1 | (*a*) Indicate the study’s design with a commonly used term in the title or the abstract | Abstract | During winter 2023, the authors conducted a cross-sectional analysis of the IQVIA Longitudinal Prescription Database… |
|  |  | (*b*) Provide in the abstract an informative and balanced summary of what was done and what was found | Abstract | During winter 2023, the authors conducted a cross-sectional analysis of the IQVIA Longitudinal Prescription Database, which reports 92% of prescriptions dispensed in U.S. retail pharmacies. The authors calculated the monthly dental opioid dispensing rate, defined as the monthly number of dispensed opioid prescriptions from dentists per 100,000 U.S. individuals, during January 2016-February 2020 and June 2020-December 2022. To prevent distortions in trends, data from March–May 2020, when dental opioid dispensing declined sharply, were excluded. Using linear segmented regression models, the authors assessed for level and slope changes in the dental opioid dispensing rate during June 2020.  **RESULTS**: Analyses included 81,189,605 dental opioid prescriptions. The annual number of prescriptions declined from 16,105,634 in 2016 to 8,910,437 in 2022 (-44.7%). During January 2016-February 2020, the dental opioid dispensing rate declined -3.9 (95% CI: -4.3, -3.6) per month. In June 2020, this rate abruptly increased by 31.4 (95% CI: 19.3, 43.5) and the monthly decline in the dental opioid dispensing rate slowed to -2.1 (95% CI: -2.6, -1.6) per month. As a result, 6.1 million more dental opioid prescriptions were dispensed during June 2020-December 2022 than would be predicted had trends during January 2016-February 2020 continued. |
| Introduction | | | |  |
| Background/rationale | 2 | Explain the scientific background and rationale for the investigation being reported | 3 | National data indicate that the rate of U.S. dental opioid prescribing per capita increased until 2016 and subsequently decreased through 2019, potentially owing to dental opioid stewardship initiatives and heightened societal awareness regarding the harms of opioids. However, more recent national data are unavailable. This is an important gap because opioid prescribing patterns by dentists may have changed since 2019. For example, the outbreak of COVID-19 in March 2020 was associated with an initial decrease in the number of dental procedures, which would tend to decrease the population-level rate of exposure to dental opioid prescriptions. On the other hand, dentists may have been more likely to prescribe opioids after procedures during the pandemic owing to barriers to scheduling routine care and follow-up visits for uncontrolled pain, which would tend to increase the population-level rate of exposure to dental opioid prescriptions. |
| Objectives | 3 | State specific objectives, including any prespecified hypotheses | 4 | Understanding recent trends in dental opioid prescribing is crucial to determine the degree to which dental opioid stewardship initiatives should still be prioritized. In this study, the authors analyzed national prescription dispensing data from January 2016 to December 2022 to assess trends in the rate of dispensed dental opioid prescriptions to U.S. patients, both overall and among key demographic subgroups. |
| Methods | | | |  |
| Study design | 4 | 4 | 2 (Line 31) | During winter 2023, the authors conducted a cross-sectional analysis of the 2016-2022 IQVIA Longitudinal Prescription Database, which contains information on 92% of prescriptions dispensed from U.S. retail pharmacies across all payers. |
| Setting | 5 | Describe the setting, locations, and relevant dates, including periods of recruitment, exposure, follow-up, and data collection | 4 | During winter 2023, the authors conducted a cross-sectional analysis of the 2016-2022 IQVIA Longitudinal Prescription Database, which contains information on 92% of prescriptions dispensed from U.S. retail pharmacies across all payers. |
| Participants | 6 | (*a*) *Cohort study*—Give the eligibility criteria, and the sources and methods of selection of participants. Describe methods of follow-up  *Case-control study*—Give the eligibility criteria, and the sources and methods of case ascertainment and control selection. Give the rationale for the choice of cases and controls  ***Cross-sectional study*—Give the eligibility criteria, and the sources and methods of selection of participants** | 4 | Analyses included opioid prescriptions written by general dentists, dental subspecialists, or oral and maxillofacial surgeons to patients residing in one of the 50 U.S. states or the District of Columbia. Prescriptions with invalid or missing data for days supplied, dispensed quantity, or strength were excluded. |
|  |  | (*b*) *Cohort study*—For matched studies, give matching criteria and number of exposed and unexposed  *Case-control study*—For matched studies, give matching criteria and the number of controls per case | N/A | N/A |
| Variables | 7 | Clearly define all outcomes, exposures, predictors, potential confounders, and effect modifiers. Give diagnostic criteria, if applicable | 5 | The primary outcome was the monthly dental opioid dispensing rate… |
| Data sources/ measurement | 8* | For each variable of interest, give sources of data and details of methods of assessment (measurement). Describe comparability of assessment methods if there is more than one group | 4 | During winter 2023, the authors conducted a cross-sectional analysis of the 2016-2022 IQVIA Longitudinal Prescription Database, which contains information on 92% of prescriptions dispensed from U.S. retail pharmacies across all payers. |
| Bias | 9 | Describe any efforts to address potential sources of bias | 5-6 | The authors assessed changes in trends in the monthly dental opioid dispensing rate between two periods: January 2016-February 2020 and June 2020-December 2022. To avoid distortions in analyses of trends, data from March-May 2020, a period during which the dental opioid dispensing date declined sharply owing to pandemic-related postponement of dental procedures, were excluded. |
| Study size | 10 | Explain how the study size was arrived at | 6 | The sample initially included 84,080,453 dental opioid prescriptions… After these exclusions, the sample included 81,189,605 dental opioid prescriptions for 53,182,495 patients. |

Continued on next page

| Quantitative variables | 11 | Explain how quantitative variables were handled in the analyses. If applicable, describe which groupings were chosen and why | 5 | The primary outcome was the monthly dental opioid dispensing rate… |
| --- | --- | --- | --- | --- |
| Statistical methods | 12 | (*a*) Describe all statistical methods, including those used to control for confounding | 5-6 | The authors assessed changes in trends in the monthly dental opioid dispensing rate… Analyses used two-sided hypothesis test with α = 0.05 and were conducted using SAS 9.4, Stata 17.1/SE, and R (version 4.2.2). |
|  |  | (*b*) Describe any methods used to examine subgroups and interactions | 5 | This outcome was calculated overall and among subgroups… and specialty (oral and maxillofacial surgeon versus general dentist or dental subspecialist). |
|  |  | (*c*) Explain how missing data were addressed | 5 | Prescriptions with invalid or missing data for days supplied, dispensed quantity, or strength were excluded. |
|  |  | (*d*) *Cohort study*—If applicable, explain how loss to follow-up was addressed  *Case-control study*—If applicable, explain how matching of cases and controls was addressed  *Cross-sectional study*—If applicable, describe analytical methods taking account of sampling strategy | N/A | N/A |
|  |  | (*e*) Describe any sensitivity analyses | N/A | N/A |
| Results | | | | |
| Participants | 13* | (a) Report numbers of individuals at each stage of study—eg numbers potentially eligible, examined for eligibility, confirmed eligible, included in the study, completing follow-up, and analysed | 6 | The sample initially included 84,080,453 dental opioid prescriptions… After these exclusions, the sample included 81,189,605 dental opioid prescriptions for 53,182,495 patients. |
|  |  | (b) Give reasons for non-participation at each stage | 6 | The sample initially included 84,080,453 dental opioid prescriptions… After these exclusions, the sample included 81,189,605 dental opioid prescriptions for 53,182,495 patients. |
|  |  | (c) Consider use of a flow diagram | N/A | N/A |
| Descriptive data | 14* | (a) Give characteristics of study participants (eg demographic, clinical, social) and information on exposures and potential confounders | 6, Table 1 | Characteristics of these patients are shown in Table 1. |
|  |  | (b) Indicate number of participants with missing data for each variable of interest | 6 | The sample initially included 84,080,453 dental opioid prescriptions for 54,699,674 patients. The authors excluded 966,804 (1.1%) prescriptions owing to missing or invalid dosing data or residence outside of one of the 50 U.S. states or the District of Columbia. |
|  |  | (c) *Cohort study*—Summarise follow-up time (eg, average and total amount) | N/A | N/A |
| Outcome data | 15* | *Cohort study*—Report numbers of outcome events or summary measures over time | N/A | N/A |
|  |  | *Case-control study—*Report numbers in each exposure category, or summary measures of exposure | N/A | N/A |
|  |  | *Cross-sectional study—*Report numbers of outcome events or summary measures | 8 | In 2016, 16,105,634 dental opioid prescriptions were dispensed, compared with 8,910,437 in 2022, representing a 44.7% decline. As shown in **Figure 1**, the rate of decline varied over time. During January 2016-February 2020, the monthly dental opioid dispensing rate declined -3.9 (95% CI: -4.3,-3.6) per month. This rate increased abruptly by 31.4 (95% CI: 19.3, 43.5) in June 2020. From June 2020-December 2022, the monthly rate of decline in the dental opioid dispensing rate slowed to -2.1 (95% CI: -2.6,-1.6) per month. During this latter period, 25.0 million dental opioid prescriptions were dispensed, but if trends from January 2016-February 2020 had continued, this number would have been 18.9 million. Consequently, 6.1 million more dental opioid prescriptions were dispensed during June 2020-December 2022 than was expected, representing a 32.0% increase (**Table 2**). |
| Main results | 16 | (*a*) Give unadjusted estimates and, if applicable, confounder-adjusted estimates and their precision (eg, 95% confidence interval). Make clear which confounders were adjusted for and why they were included | 8 | In 2016, 16,105,634 dental opioid prescriptions were dispensed, compared with 8,910,437 in 2022, representing a 44.7% decline. As shown in **Figure 1**, the rate of decline varied over time. During January 2016-February 2020, the monthly dental opioid dispensing rate declined -3.9 (95% CI: -4.3,-3.6) per month. This rate increased abruptly by 31.4 (95% CI: 19.3, 43.5) in June 2020. From June 2020-December 2022, the monthly rate of decline in the dental opioid dispensing rate slowed to -2.1 (95% CI: -2.6,-1.6) per month. During this latter period, 25.0 million dental opioid prescriptions were dispensed, but if trends from January 2016-February 2020 had continued, this number would have been 18.9 million. Consequently, 6.1 million more dental opioid prescriptions were dispensed during June 2020-December 2022 than was expected, representing a 32.0% increase (**Table 2**). |
|  |  | (*b*) Report category boundaries when continuous variables were categorized | N/A | N/A |
|  |  | (*c*) If relevant, consider translating estimates of relative risk into absolute risk for a meaningful time period | N/A | N/A |

Continued on next page

| Other analyses | 17 | Report other analyses done—eg analyses of subgroups and interactions, and sensitivity analyses | 12 | As in the main analysis, there was a level increase and slope increase (i.e., slowing of the pre-existing decline) during June 2020 in each age group, except for patients aged 12-25 years, for which there was neither a level nor a slope change **(Appendix 2)**. There was also heterogeneity in changes during June 2020 by method of payment **(Appendix 3)**. |
| --- | --- | --- | --- | --- |
| Discussion | | | | |
| Key results | 18 | Summarise key results with reference to study objectives | 13 | To the authors’ knowledge, this study provides the most recent national data on trends in U.S. dental opioid prescribing and is the first to capture changes in this prescribing during the COVID-19 pandemic. Findings indicate that the annual number of dispensed dental opioid prescriptions declined 44.7% between 2016 and 2022. However, the rate of decline varied substantially over time. The sharpest decline in the dental opioid dispensing rate occurred between January 2016 and February 2020. After June 2020, this rate continued to decline, but at a slower pace than before June 2020. As a result of this slowing, 6.1 million more dental opioid prescriptions were dispensed during June 2020-December 2022 than would be predicted had trends from January 2016-February 2020 continued. |
| Limitations | 19 | Discuss limitations of the study, taking into account sources of potential bias or imprecision. Discuss both direction and magnitude of any potential bias | 15 | However, the study also had limitations… |
| Interpretation | 20 | Give a cautious overall interpretation of results considering objectives, limitations, multiplicity of analyses, results from similar studies, and other relevant evidence | 16 | Opioid prescribing by U.S. dentists decreased markedly between 2016 and 2022, but the rate of decline has slowed substantially since June 2020. Renewed investment in opioid stewardship efforts is needed to ensure that the contribution of dental opioid prescribing to the U.S. opioid epidemic continues to be mitigated. |
| Generalisability | 21 | Discuss the generalisability (external validity) of the study results | 15 | The primary strength of this study was the use of timely data from an all-payer national database. |
| Other information | |  | | |
| Funding | 22 | Give the source of funding and the role of the funders for the present study and, if applicable, for the original study on which the present article is based | Title page | This study was funded by a grant from the Benter Foundation… The funders played no role in the design and conduct of the study; collection, management, analysis, and interpretation of the data; preparation, review, or approval of the manuscript; and decision to submit the manuscript for publication. |

*Give information separately for cases and controls in case-control studies and, if applicable, for exposed and unexposed groups in cohort and cross-sectional studies.

**Note:** An Explanation and Elaboration article discusses each checklist item and gives methodological background and published examples of transparent reporting. The STROBE checklist is best used in conjunction with this article (freely available on the Web sites of PLoS Medicine at http://www.plosmedicine.org/, Annals of Internal Medicine at http://www.annals.org/, and Epidemiology at http://www.epidem.com/). Information on the STROBE Initiative is available at www.strobe-statement.org.
