# Supplementary material for: Association between the COVID-19 outbreak and opioid prescribing by U.S. dentists
Source: PLoS One. 2023 Nov 2;18(11):e0293621. doi: 10.1371/journal.pone.0293621 (PMC10621808; doi:10.1371/journal.pone.0293621)

**S1 Fig.** Monthly dental opioid dispensing rate by age group, 2016 – 2022.

Graphs for patients aged 0-11 years are not displayed owing to small sample sizes.


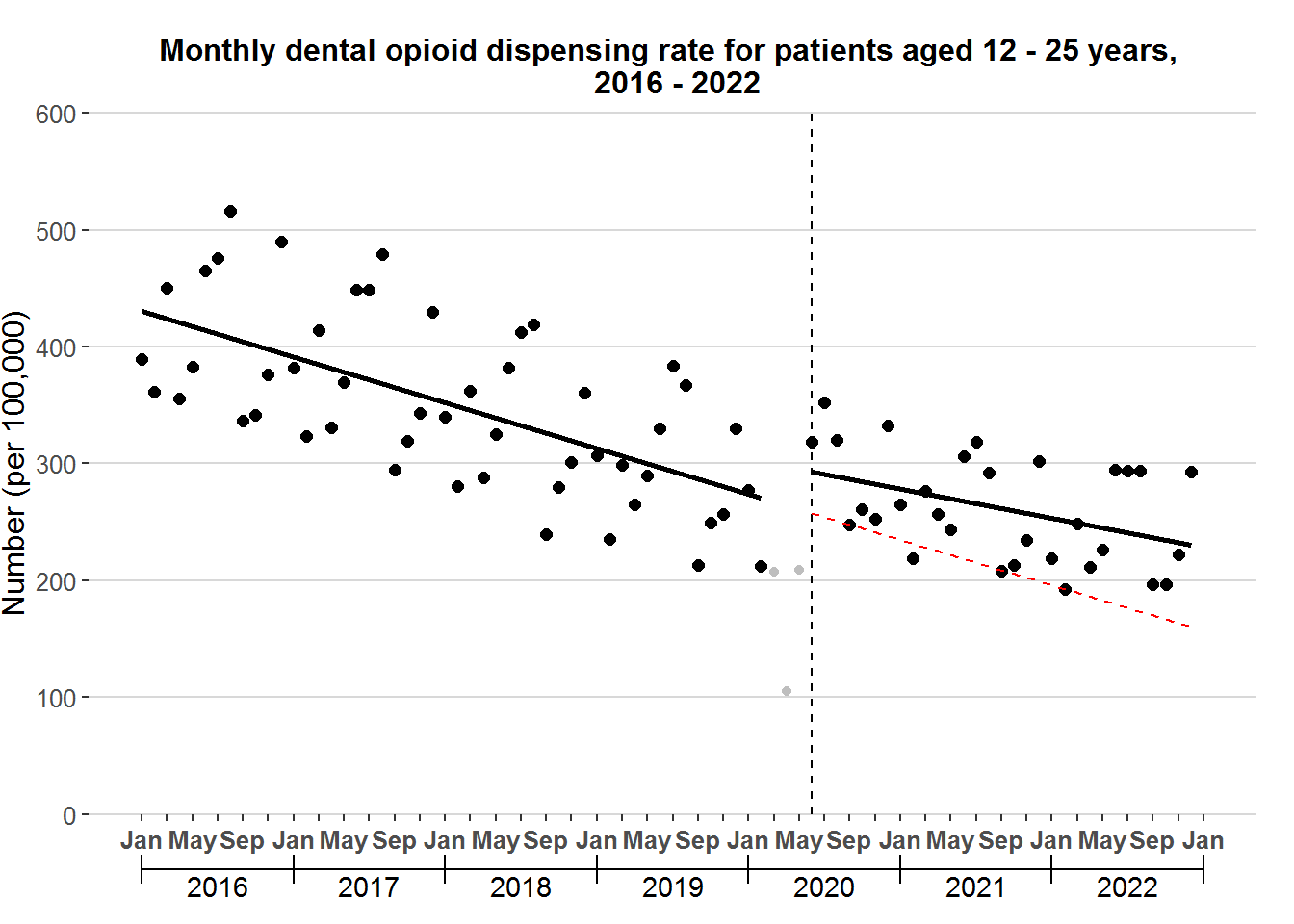


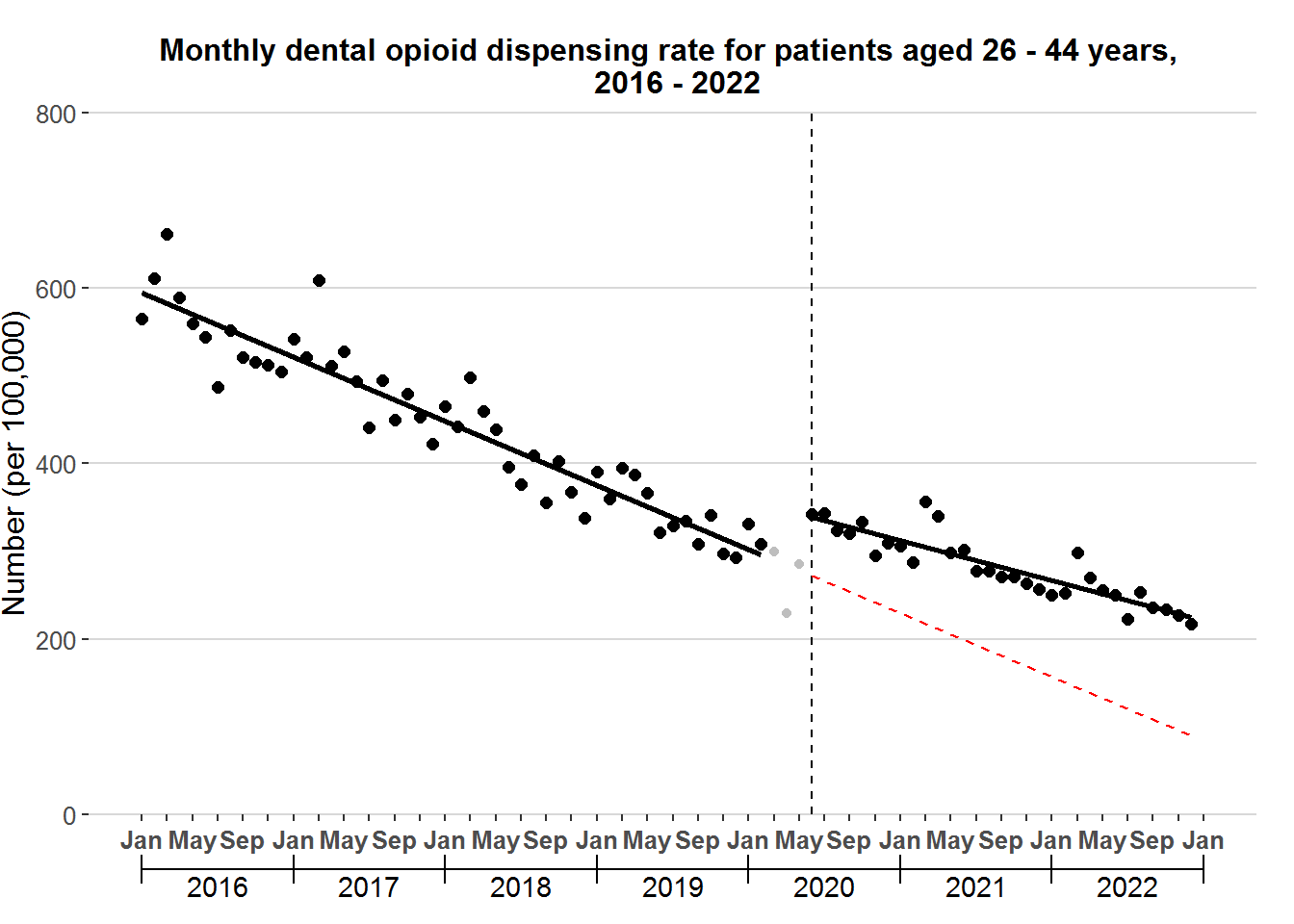


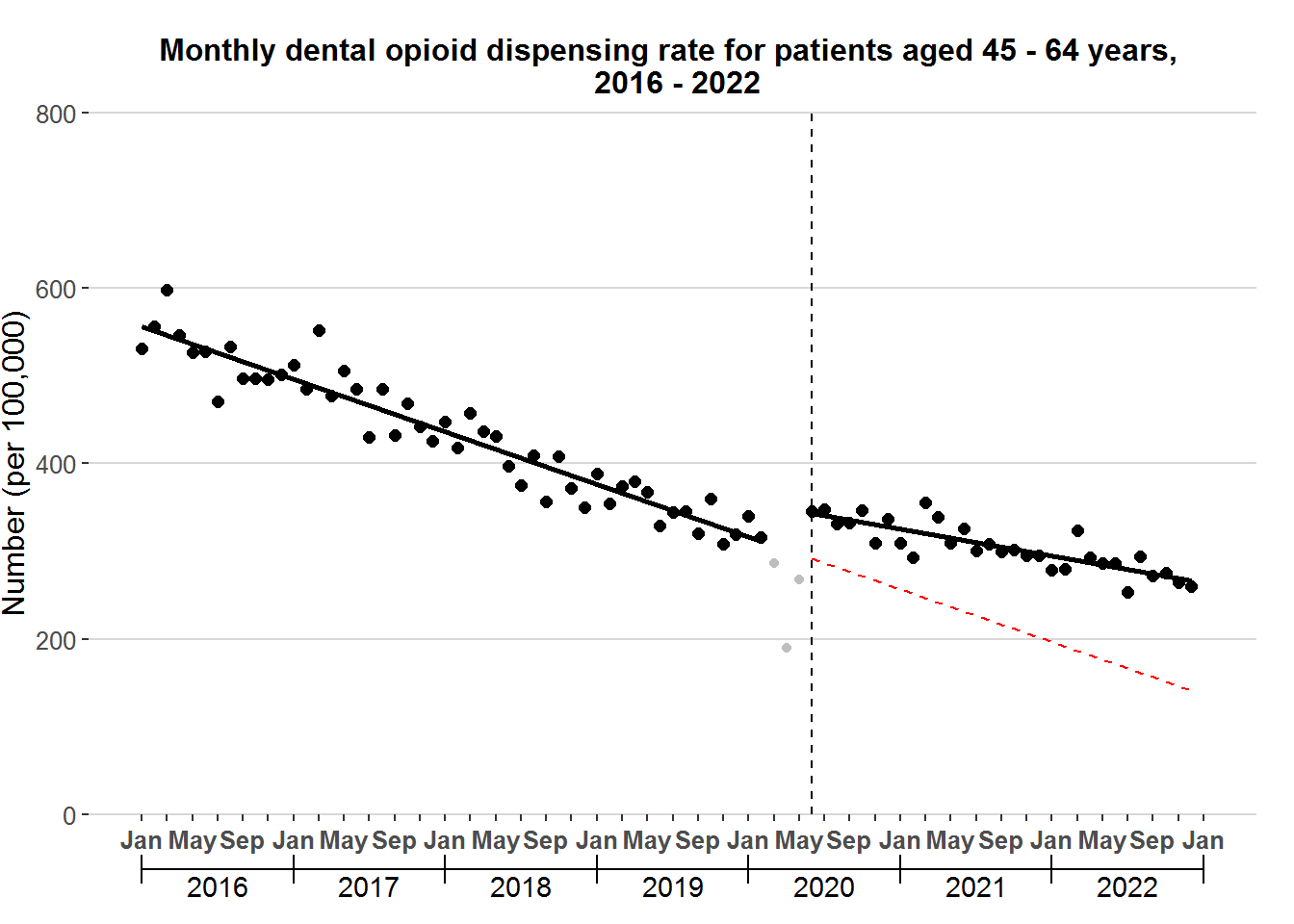


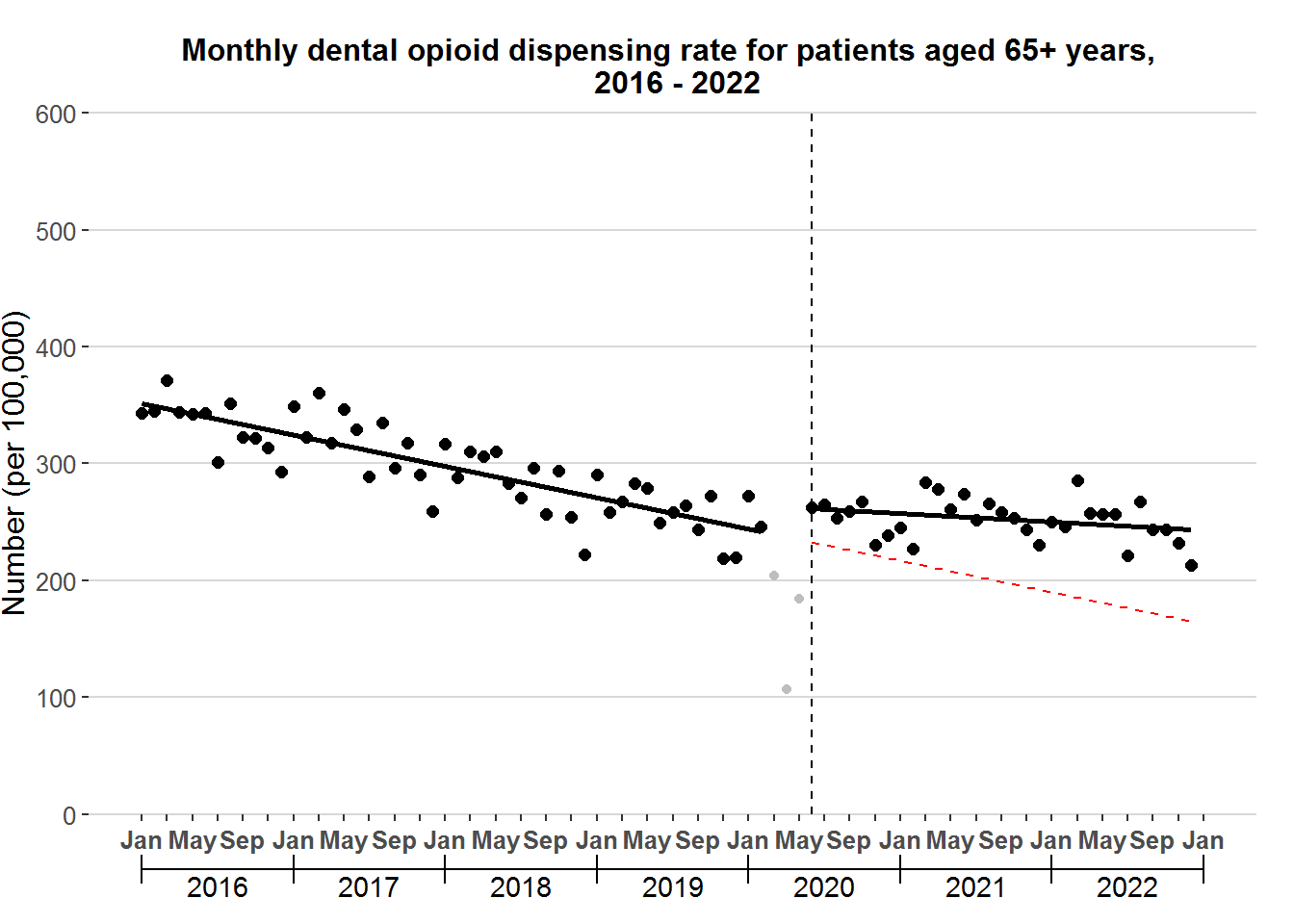

Supplement: S1 Fig — This rate is defined as the monthly number of dispensed opioid prescriptions from dentists per 100,000 U.S. individuals of all ages. The black lines are fitted lines from segmented regression models assessing for abrupt level or slope changes in June 2020 (vertical line). The red dashed line is the counterfactual trend, representing the trend that would have occurred had trends from January 2016-February 2020 continued. (DOCX) [file pone.0293621.s002.docx]
