# Supplementary material for: Association between the COVID-19 outbreak and opioid prescribing by U.S. dentists
Source: PLoS One. 2023 Nov 2;18(11):e0293621. doi: 10.1371/journal.pone.0293621 (PMC10621808; doi:10.1371/journal.pone.0293621)
Supplement: S1 Table — (DOCX) [file pone.0293621.s009.docx]

**S1 Table.** Opioids in the IQVIA data, number of prescriptions (% of total in year).

| **Opioid Type** | **2016** | **2017** | **2018** | **2019** | **2020** | **2021** | **2022** |
| --- | --- | --- | --- | --- | --- | --- | --- |
| Buprenorphine | 212 (0.0%) | 215 (0.0%) | 138 (0.0%) | 70 (0.0%) | 29 (0.0%) | 34 (0.0%) | 36 (0.0%) |
| Butorphanol | 214 (0.0%) | 136 (0.0%) | 123 (0.0%) | 83 (0.0%) | 56 (0.0%) | 61 (0.0%) | 64 (0.0%) |
| Codeine | 3,268,887 (20.3%) | 3,158,894 (21.4%) | 2,919,549 (23.1%) | 2,728,951 (24.9%) | 2,036,981 (25.7%) | 2,374,786 (24.1%) | 2,059,647 (23.1%) |
| Dihydrocodeine | 52 (0.0%) | 68 (0.0%) | 36 (0.0%) | 22 (0.0%) | 13 (0.0%) | 13 (0.0%) | 15 (0.0%) |
| Fentanyl | 1,312 (0.0%) | 1,040 (0.0%) | 757 (0.0%) | 390 (0.0%) | 200 (0.0%) | 233 (0.0%) | 200 (0.0%) |
| Hydrocodone | 10,125,988 (62.9%) | 9,203,600 (62.2%) | 7,676,481 (60.7%) | 6,457,514 (58.9%) | 4,574,944 (57.8%) | 5,822,251 (59.1%) | 5,302,876 (59.5%) |
| Hydromorphone | 12,369 (0.1%) | 10,499 (0.1%) | 7,829 (0.1%) | 6,187 (0.1%) | 4,757 (0.1%) | 5,814 (0.1%) | 5,073 (0.1%) |
| Levorphanol | 4 (0.0%) | 5 (0.0%) | 6 (0.0%) | 0 (0.0%) | 2 (0.0%) | 3 (0.0%) | 0 (0.0%) |
| Meperidine | 60,737 (0.4%) | 50,491 (0.3%) | 41,840 (0.3%) | 27,145 (0.2%) | 13,520 (0.2%) | 15,669 (0.2%) | 12,408 (0.1%) |
| Methadone | 885 (0.0%) | 681 (0.0%) | 578 (0.0%) | 433 (0.0%) | 221 (0.0%) | 268 (0.0%) | 242 (0.0%) |
| Morphine | 3,279 (0.0%) | 2,683 (0.0%) | 2,059 (0.0%) | 1,290 (0.0%) | 839 (0.0%) | 972 (0.0%) | 832 (0.0%) |
| Opium | 2 (0.0%) | 6 (0.0%) | 4 (0.0%) | 4 (0.0%) | 1 (0.0%) | 1 (0.0%) | 2 (0.0%) |
| Oxycodone | 1,932,536 (12.0%) | 1,660,625 (11.2%) | 1,302,670 (10.3%) | 1,070,066 (9.8%) | 769,402 (9.7%) | 1,005,052 (10.2%) | 937,821 (10.5%) |
| Oxymorphone | 293 (0.0%) | 221 (0.0%) | 133 (0.0%) | 75 (0.0%) | 18 (0.0%) | 16 (0.0%) | 20 (0.0%) |
| Pentazocine | 1,973 (0.0%) | 1,277 (0.0%) | 1,104 (0.0%) | 416 (0.0%) | 236 (0.0%) | 138 (0.0%) | 69 (0.0%) |
| Tapentadol | 1,139 (0.0%) | 911 (0.0%) | 655 (0.0%) | 331 (0.0%) | 187 (0.0%) | 198 (0.0%) | 129 (0.0%) |
| Tramadol | 695,752 (4.3%) | 697,774 (4.7%) | 689,964 (5.5%) | 672,200 (6.1%) | 514,050 (6.5%) | 634,340 (6.4%) | 591,003 (6.6%) |
| **TOTAL** | 16,105,634 (100.0%) | 14,789,126 (100.0%) | 12,643,926 (100.0%) | 10,965,177 (100.0%) | 7,915,456 (100.0%) | 9,859,849 (100.0%) | 8,910,437 (100.0%) |
