# Supplementary material for: Association between the COVID-19 outbreak and opioid prescribing by U.S. dentists
Source: PLoS One. 2023 Nov 2;18(11):e0293621. doi: 10.1371/journal.pone.0293621 (PMC10621808; doi:10.1371/journal.pone.0293621)
Supplement: S2 Table — (DOCX) [file pone.0293621.s010.docx]

**S2 Table.** Population denominators, 2016 – 2022.

For population denominators by payer type, we utilized the 2016-2021 Medical Expenditure Panel Survey (MEPS), which gives estimates in terms of the U.S. civilian population. To extrapolate the 2022 estimates, we first multiplied the 2021 denominator by 1.004, which is how much the Census estimates the civilian population grew between July 2021 and July 2022. Estimating that Medicaid enrollment grew 6.7% between 2021 and 2022 (as indicated by CMS enrollment data), this implies about 4,512,820 more beneficiaries, meaning the percentage of the population that was shared by Medicaid increased by 1.36% between 2021 and 2022 (i.e., 4,512,820 divided by 330,784,232). We assumed that this increased share was accounted for by losses in private insurance (i.e., the share of the population covered by private insurance decreased by 1.36% from 2021 to 2022), and kept the proportion of the population covered by Medicare and cash in 2022 to be the same it was in 2021 in order to calculate the final 2022 denominators.

For population denominators by age group, sex, and Census region, our study utilized U.S. Census population estimates from both the 2010 U.S. Census (2016-2019) and 2020 U.S. Census (2020-2022). To maintain consistency with the MEPS denominators, we calculated these denominators based on civilian population as well. However, the 2016-2019 Census estimates seemed to underestimate population counts. For example, the 2010 U.S. Census estimated that there were 328,297,202 U.S. civilians in July 2020, while the 2020 U.S. estimated 330,405,064 U.S. civilians in July 2020 (1.00642059 times higher). To address this discrepancy, we multiplied the 2016-2019 population estimates by the appropriate factors. The table below shows the denominators used in the analysis. Note: we used the denominators for all civilians for the specialty type subgroup analysis as well.

| **Denominator** | **2016** | **2017** | **2018** | **2019** | **2020** | **2021** | **2022** |
| --- | --- | --- | --- | --- | --- | --- | --- |
| All civilians | 323,935,861 | 326,011,345 | 327,742,152 | 329,243,484 | 330,405,064 | 330,784,232 | 331,163,835 |
| 0 – 11 years | 49,070,947 | 48,980,721 | 48,782,731 | 48,450,713 | 48,080,148 | 47,411,781 | 46,752,705 |
| 12 – 25 years | 61,149,751 | 60,817,819 | 60,507,119 | 60,318,753 | 60,126,619 | 60,072,320 | 60,018,070 |
| 26 – 44 years | 80,317,548 | 81,245,425 | 82,184,297 | 83,012,868 | 83,626,333 | 84,003,726 | 84,382,822 |
| 45 – 64 years | 85,479,365 | 85,486,210 | 85,172,410 | 84,664,405 | 84,133,668 | 83,448,452 | 82,768,817 |
| 65 years and above | 48,128,931 | 49,644,105 | 51,206,037 | 52,851,264 | 54,438,296 | 55,847,953 | 57,294,112 |
| Male | 160,070,364 | 161,125,245 | 161,988,449 | 162,729,494 | 163,300,466 | 163,460,070 | 163,619,830 |
| Female | 163,865,104 | 164,886,056 | 165,753,752 | 166,514,027 | 167,104,598 | 167,324,162 | 167,544,014 |
| Northeast | 57,702,360 | 57,722,255 | 57,723,945 | 57,639,860 | 57,482,153 | 57,114,721 | 56,749,638 |
| Midwest | 68,550,567 | 68,693,913 | 68,794,698 | 68,872,499 | 68,848,928 | 68,753,873 | 68,658,949 |
| South | 121,542,858 | 122,745,273 | 123,780,853 | 124,816,396 | 125,790,880 | 126,600,242 | 127,414,812 |
| West | 76,192,650 | 76,889,002 | 77,469,846 | 77,928,739 | 78,283,103 | 78,315,396 | 78,347,702 |
| Cash | 24,608,684 | 21,497,527 | 21,240,330 | 20,614,305 | 21,390,906 | 21,166,126 | 21,250,791 |
| Medicaid | 58,410,869 | 58,740,968 | 57,595,895 | 59,880,715 | 59,152,258 | 67,355,521 | 72,137,763 |
| Medicare | 50,581,241 | 51,817,805 | 53,791,802 | 53,991,728 | 57,248,595 | 57,368,268 | 57,597,741 |
| Commercial | 189,540,892 | 192,723,609 | 193,699,861 | 192,909,946 | 190,753,538 | 185,359,478 | 181,588,096 |
