# Supplementary material for: Association between the COVID-19 outbreak and opioid prescribing by U.S. dentists
Source: PLoS One. 2023 Nov 2;18(11):e0293621. doi: 10.1371/journal.pone.0293621 (PMC10621808; doi:10.1371/journal.pone.0293621)
Supplement: S3 Table — (DOCX) [file pone.0293621.s011.docx]

**S3 Table.** Segmented regression model coefficients for mean total MME and mean days supplied per dental opioid prescription.

|  | **Segmented regression model coefficients, number [95% CI]** | | | | | |
| --- | --- | --- | --- | --- | --- | --- |
|  | **Intercept** | **Change per month before February 2020** | **Level change during June 2020** | **Slope change after June 2020** | **Change per month after June 2020** | **Observed minus expected # in Dec 2022 (% difference)** |
|  |  |  |  |  |  |  |
| **Mean total MME** | 118.6 [116.8, 120.4] | -0.69 [-0.74, -0.64] | 0.71 [-0.42, 1.83] | 0.5 [0.45, 0.55] | -0.2 [-0.21, -0.18] | 15.6 (25.0%) |
|  |  |  |  |  |  |  |
| **Mean days supplied** | 3.6 [3.6, 3.6] | -0.01 [-0.01, -0.01] | 0 [-0.02, 0.01] | 0.01 [0.01, 0.01] | 0 [0, 0] | 0.2 (7.9%) |
